# Six-Month Follow-Up of Immune Responses after a Rapid Mass Vaccination against SARS-CoV-2 with BNT162b2 in the District of Schwaz/Austria

**DOI:** 10.3390/v14081642

**Published:** 2022-07-27

**Authors:** Zoltán Bánki, Lisa Seekircher, Barbara Falkensammer, David Bante, Helena Schäfer, Teresa Harthaller, Janine Kimpel, Peter Willeit, Dorothee von Laer, Wegene Borena

**Affiliations:** 1 Institute of Virology, Medical University of Innsbruck, 6020 Innsbruck, Austria; zoltan.banki@i-med.ac.at (Z.B.); barbara.falkensammer@i-med.ac.at (B.F.); david.bante@i-med.ac.at (D.B.); helena.schäfer@i-med.ac.at (H.S.); teresa.harthaller@i-med.ac.at (T.H.); janine.kimpel@i-med.ac.at (J.K.); dorothee.von-laer@i-med.ac.at (D.v.L.); 2 Clinical Epidemiology Team, Medical University of Innsbruck, 6020 Innsbruck, Austria; lisa.seekircher@i-med.ac.at (L.S.); peter.willeit@i-med.ac.at (P.W.); 3 Department of Public Health and Primary Care, University of Cambridge, Cambridge CB2 1TN, UK

**Keywords:** SARS-CoV-2, COVID-19, vaccination, T cell response, antibody response

## Abstract

In response to a large outbreak of the SARS-CoV-2 Beta (B.1.351) variant in the district Schwaz, Austria, a rapid mass vaccination campaign with BNT162b2 was carried out in spring 2021, immunizing more than 70% of the adult population within one week. Subsequent analysis revealed that the mass vaccination was associated with a significant reduction in new SARS-CoV-2 infections compared to control districts. Here, we aimed to evaluate both SARS-CoV-2-specific T- and B-cell responses at 35 ± 8 and 215 ± 7 days after the second dose in 600 study subjects who participated at both time points. Overall, a robust antibody and T-cell response was measured at day 35, which waned over time. Nevertheless, all persons preserved seropositivity and T cell response could still be detected in about half of the participants at day 215. Further, antibody response correlated negatively with age; however, in persons who experienced SARS-CoV-2 infection prior to study enrolment, the serum levels of both S- and N-specific antibodies surprisingly increased with age. In contrast, there was no correlation of T cell response with age. We could not detect any sex-related difference in the immune responses. SARS-CoV-2 infections prior to study enrolment or incident infections before day 215 resulted in higher antibody levels and T cell responses at day 215 compared to study participants with no history of infection. Collectively, our data support that vaccination with BNT162b2 against COVID-19 provides a durable immune response and emphasize the usefulness of vaccination even after a natural infection.

## 1. Introduction

The BNT162b2 mRNA vaccine by BioNTech/Pfizer is one of the successful vaccinations against SARS-CoV-2 developed in response to the COVID-19 pandemic [1]. Early studies on BNT162b2 showed excellent safety and immunogenicity [2,3,4]. Despite the observed gradual decline in the efficacy of preventing symptomatic infections over 6 months, BNT162b2 vaccination is highly efficient in preventing severe COVID-19 [1]. The decline in BNT162b2 vaccine efficacy suggests a waning of vaccine-induced immunity over time. Indeed, studies investigating the kinetics and persistence of cellular and humoral immune responses to the BNT162b2 vaccine all observed a gradual decrease in SARS-CoV-2-specific immunity within 6 months [5,6,7,8,9].

The emergence of new SARS-CoV-2 variants of concern (VoC) represents a continuous challenge. One of the largest outbreaks of Beta and Alpha variants in Europe occurred in the district Schwaz, Austria, in spring 2021. In response to the situation, the Government of Austria with the support of BioNTech/Pfizer organized a rapid mass vaccination campaign using 100,000 extra vaccine doses available for the entire adult population in the district Schwaz. As expected, a subsequent analysis of the impact of this mass vaccination revealed a reduction in incident infections as well as hospital and intensive care unit admissions due to SARS-CoV-2 in the Schwaz district compared to control districts [10]. This and a number of other studies clearly demonstrate the importance of successful vaccination campaigns in fighting the COVID-19 pandemic even if vaccinations are not fully protective against acquiring infections and only reduce severe disease outcomes, especially for the immune escape variants of concern [11,12,13,14,15].

There is a growing body of data on the efficacy, immunogenicity and kinetics of the immune response over time following vaccination against SARS-CoV-2, but there is not yet a complete picture of the duration of immunity, particularly with regard to the T-cell response. Therefore, we conducted the Shieldvacc-2 study, a phase 4 open-label clinical trial, among individuals vaccinated with two doses of BNT162b2 in the district Schwaz. In addition to the primary objectives of evaluating the predictive values and the correlation of immune parameters with the relative risk of incident SARS-CoV-2 infections [16], we analyzed immune parameters at day 35 ± 8 (D35) and day 215 ± 7 (D215) after receiving the second doses of BNT162b2 in a cohort of individuals participating at both time points.

## 2. Materials and Methods

### 2.1. Study Design and Population

The Shieldvacc-2 study, a phase 4 open-label clinical trial, was approved by the ethics committee of the Medical University of Innsbruck (no. 1168/2021) and has been registered at the European Union Drug Regulating Authorities Clinical Trials Database (EudraCT number: 2021-002030-16). Written informed consent was provided by study participants or—if appropriate—by the individual’s legal or custodial representative. Individuals were eligible for inclusion if they (i) were aged 16 years or older; (ii) had been vaccinated with two 30 µg doses of BNT162b2 delivered by intramuscular injection, with the second dose having been administered 35 ± 8 days before study enrolment; (iii) understood and agreed to comply with the study procedures; and (iv) were willing to be contacted by telephone and to complete a diary during study participation in an online participant portal. The following exclusion criteria were applied: (i) prior administration of an investigational coronavirus (SARS-CoV, MERS-CoV) vaccine or current/planned simultaneous participation in another interventional study to either prevent or treat COVID-19; (ii) a contraindication to blood draws (e.g., bleeding disorders); (iii) participation in an interventional clinical study within 30 days prior to study inclusion.

At the baseline between 15 and 21 May of 2021, blood samples were collected in S-Monovette tubes (Sarstedt, Nümbrecht, Germany) containing ethylenediaminetetraacetic acid anticoagulant (EDTA KE/9 mL) for subsequent analysis of SARS-CoV-2-specific humoral responses. Furthermore, in order to evaluate cellular immune responses, in a random subgroup of 929 participants, additional blood samples were collected in S-Monovette tubes (Sarstedt, Nümbrecht, Germany) containing lithium-heparin anticoagulant (Li-Heparin LH/9 mL). Six months after the study baseline between 11 and 18 November of 2021, participants were again invited for blood draws. A total of 600 individuals provided blood samples for analysis of both humoral and cellular immune responses at both time points and were included in this study.

### 2.2. Serological Assays for Measuring SARS-CoV-2 Spike- and Nucleoplasmid-Specific IgGs

For the qualitative and quantitative determinations of SARS-CoV-2 Spike (S)-specific IgG, we used the assay SARS-CoV-2 IgG II Quant running fully automated on Abbott Alinity immunoassay system (Abbott Park, IL, USA). SARS-CoV-2 IgG II Quant is a chemiluminescent microparticle immunoassay (CMIA) in which the microparticles are coated with spike-receptor-binding domain (RBD) specific to SARS-CoV-2. Light emitted from acridinium-labeled anti-human IgG antibodies is measured as relative light units (RLUs). By using six standards (calibrators) of known concentrations, the system calculates sample antibody concentrations based on the Four Parameter Logistic (4PL) regression method. Results are provided in binding antibody units per milliliter (BAU/mL) and values above 7.1 BAU/mL are interpreted as positive.

For the detection of nucleocapsid (N)-specific immunoglobulins (Ig), plasma samples were further analyzed using the assay Elecsys Anti-SARS-CoV-2 performed on Roche cobas e 411 analyzer (Roche Diagnostics, Indianapolis, USA) according to the manufacturer’s instructions. Elecsys Anti-SARS-CoV-2 is an electroluminescence immunoassay (ECLIA) intended for the qualitative detection of SARS-CoV-2-specific antibodies. A voltage released to the Ruthenylated SARS-CoV-2 antigen–antibody complex, which in turn is bound to an electrode, results in chemiluminescent emission, which is measured by a photomultiplier. Results are presented as cutoff index (COI) and values ≥ 1.0 are interpreted as positive.

### 2.3. QuantiFERON Interferon-γ Release Assay (IGRA)

Cellular immune responses were evaluated by QuantiFERON (QFN) SARS-CoV-2 Interferon-Gamma (IFN-γ) release assay (IGRA) (Qiagen, Hilden, Germany). Blood samples were collected in S-Monovette tubes (Sarstedt, Nümbrecht, Germany) containing lithium-heparin anticoagulant (Li-Heparin LH/9ml), stored at room temperature (17–25 °C), and processed within ≤8 h at the laboratory of the Institute of Virology of the Medical University of Innsbruck, Austria. An amount of 1 mL whole blood was loaded onto QFN tubes coated with two spike-derived peptide antigen pools (Ag1 and Ag2), and including QFN tubes for a negative control (Nil) and for a positive control (Mitogen). Ag1 contains CD4, whereas Ag2 contains CD4/CD8 peptide pools derived from SARS-CoV-2 spike (S1 S2 RBD) antigen. After 16–24 h of incubation at 37 °C, tubes were centrifuged for 15 min at 3.000 g, plasma was harvested, stored at −80 °C until use and IFN-γ was measured by QFN human IFN-γ ELISA Kit (Qiagen, Hilden, Germany) according to the manufacturer’s instructions using an Epoch Microplate Spectrophotometer (BioTek, Winooski, VT, USA). The Qiagen QuantiFERON SARS-CoV-2 IGRA was dedicated for research use only, no performances and official cut-off value have yet been defined by the manufacturer at the time of our study. Therefore, the ratio of IFN-γ values from SARS-CoV-2-specific stimulation and the unstimulated control was determined as the stimulation index (SI) to mitigate against the background IFN-γ in the sample. We considered samples with SI values < 2 as negative, 2 ≤ SI < 3 as weakly reactive and values ≥ 3 as reactive.

### 2.4. Statistics

**Figure 1:**
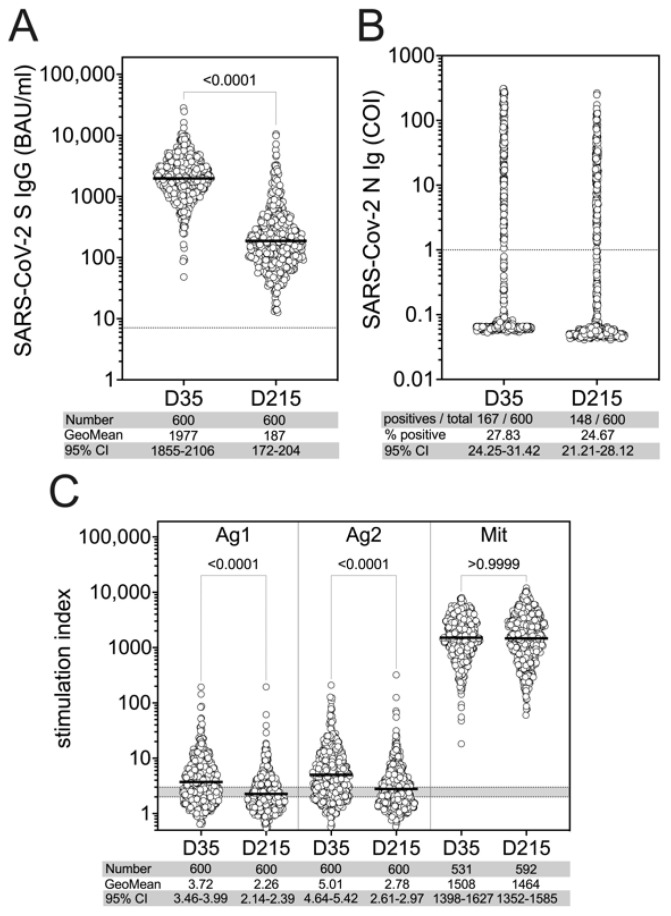
SARS-CoV-2-specific immune responses at D35 and D215 after the second vaccination with BNT162b2. Serum levels of SARS-CoV-2 S- (**A**) and N-specific (**B**) antibodies are shown in samples collected at day 35 and day 215 (D35 and D215). T cell response (**C**) is presented as the stimulation index (SI) calculated as the ratios of IFN-γ values from SARS-CoV-2-specific Ag1 (CD4 peptide pool) and Ag2 (CD4/CD8 peptide pool) as well as mitogen (Mit) stimulations and the respective unstimulated control. Differences of immune parameters between baseline and follow-up were tested by applying non-parametric paired Wilcoxon test (**A**,**B**) or non-parametric Kruskal–Wallis test followed by Dunn’s multiple comparisons (**C**).

**Figure 2:**
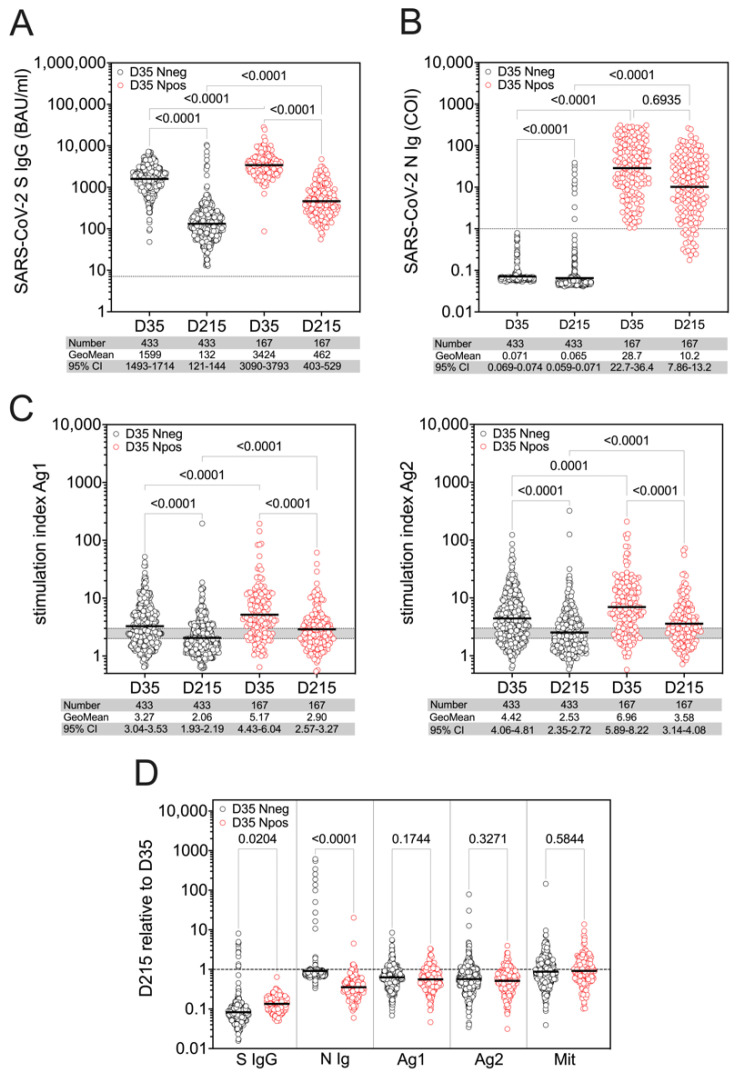
Levels and persistence of SARS-CoV-2-specific immune responses after vaccination in individuals convalescent from SARS-CoV-2 infection (D35 Npos, red symbols) or not (D35 Nneg, black symbols) prior to vaccination. Serum levels of SARS-CoV-2 S- (**A**) and N-specific (**B**) antibodies are shown in samples collected at D35 and D215. T cell response (**C**) is presented as the stimulation index (SI) for SARS-CoV-2-specific Ag1 and Ag2. (**D**) Relative response in D35 Nneg and Npos groups for S IgG, N Ig, Ag1, Ag2 and Mit calculated from values measured at D215 relative to D35. Differences of immune parameters between baseline and follow-up and between individuals with and without prior SARS-CoV-2 infections were tested by applying non-parametric Kruskal–Wallis test followed by Dunn’s multiple comparisons.

**Figure 3:**
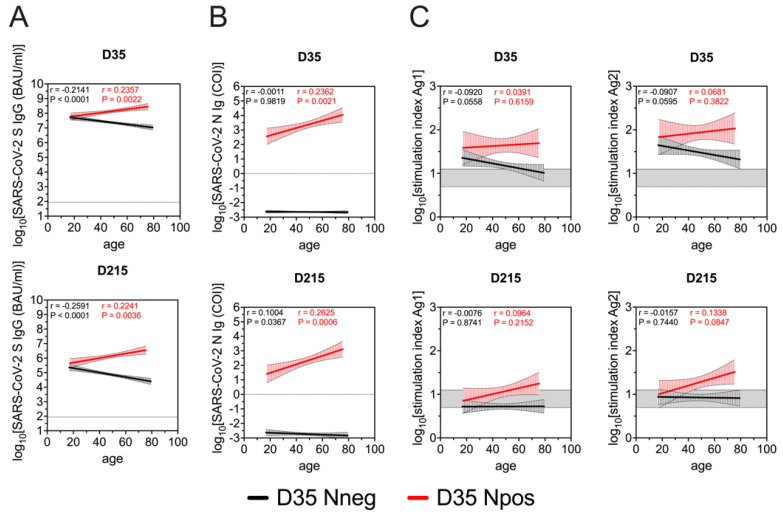
Correlation between age and SARS-CoV-2-specific S IgG (**A**), N Ig (**B**) and T cell (**C**) responses after BNT162b2 vaccination in groups with no (D35 Nneg, black) and prior SARS-CoV-2 infection (D35 Npos, red). Pearson correlation coefficients were calculated for log10-transformed values of immunological parameters at different time points and age at baseline separately for individuals with and without prior SARS-CoV-2 infection.

**Figure 4:**
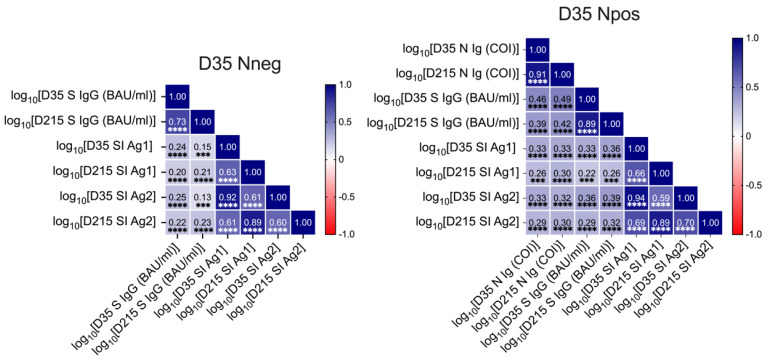
Correlation of different immune parameters measured at D35 and D215 in the D35 Nneg and Npos groups. Pearson correlation coefficients were calculated to assess the correlation between log10-transformed values of immunological parameters measured at study baseline and at follow-up. *p* values ≤ 0.001 and < 0.0001 are represented with *** and ****, respectively.

**Figure 5:**
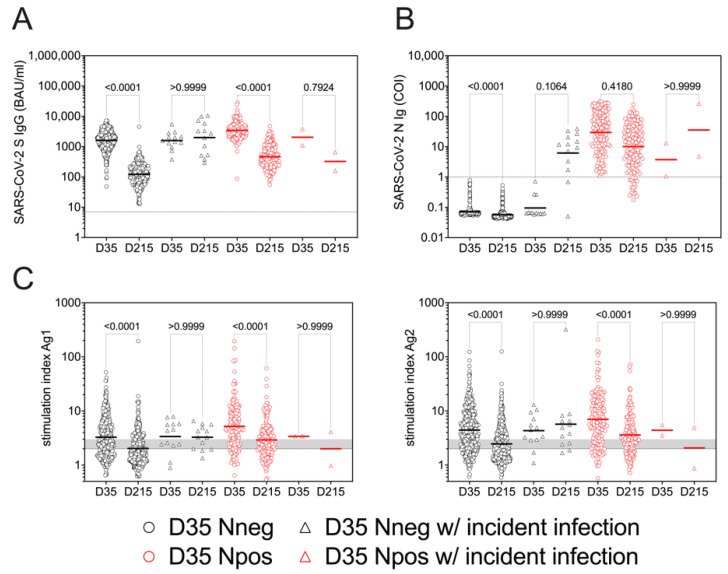
Effect of an incident infection before D215 on the vaccine induced SARS-CoV-2-specific immune response in D35 Nneg (black symbols) and Npos (red symbols) groups. Serum levels of SARS-CoV-2 S- (**A**) and N-specific (**B**) antibodies as well as T cell response represented by stimulation index (SI) to Ag1 and Ag2 (**C**) are shown in samples collected at D35 and D215 with (triangle) or without (circle) incident SARS-CoV-2 infection. Differences of immune parameters were tested by applying non-parametric Kruskal–Wallis test followed by Dunn’s multiple comparisons.

Results were analyzed in GraphPad Prism software (version 9.3.0). Because immune parameter levels were non-normally distributed, individual values were plotted on a logarithmic axis. We summarized values of humoral and cellular immune responses by the geometric mean with 95% confidence interval (CI). Differences of immune parameters between baseline and follow-up and between individuals with and without prior SARS-CoV-2 infections were tested by applying non-parametric paired Wilcoxon test (Figure 1A,B) or non-parametric Kruskal–Wallis test followed by Dunn’s multiple com-parisons (Figures 1C, 2A–D, and 5; Supplementary Figures S1 and S2). Log10-transformed values of the immune parameters were correlated with age. We estimated Pearson cor-relation coefficients of log10-transformed values of immunological parameters at differ-ent time points and age at baseline separately for individuals with and without prior SARS-CoV-2 infection (Figure 3). Furthermore, we calculated Pearson correlation coeffi-cients to assess the correlation between immunological parameters measured at study baseline and at follow-up (Figure 4). *p* values ≤ 0.05 were deemed statistically significant.

## 3. Results

Demographic characteristics of the cohort are included in Table 1. In the participants contributing to this analysis (*n* = 600), we found 100% seropositivity for SARS-CoV-2 spike-specific IgGs at 35 ± 8 (D35) and also at 215 ± 7 (D215) days after the second vaccination. The levels of SARS-CoV-2 spike-specific IgG reached a geometric mean of 1977 BAU/mL at D35, which significantly decreased to 187 BAU/mL at D215 (Figure 1A). In addition, 167 out of 600 study subjects corresponding to 27.83% had anti-SARS-CoV-2 N protein antibodies (values greater than 1.0 COI) at D35 indicating an infection prior to the sampling at D35. Detectable N seropositivity was found in 148 out of 600 individuals amounting to 24.67% at D215 (Figure 1B).

In addition to SARS-CoV-2-specific antibody responses, we evaluated SARS-CoV-2-specific T cell responses using QFN IGRA (Qiagen, Hilden, Germany) after the stimulation of heparinized whole blood with the SARS-CoV-2 spike-derived peptide pools Ag1 (CD4 peptide pool) and Ag2 (CD4/CD8 peptide pool). Compared to the IFN-γ concentration (IU/mL) measured in the negative control tubes (Nil), we found significantly elevated values after stimulation with both Ag1 and Ag2 (Supplementary Figure S1). We determined the stimulation index (SI) by calculating ratios of IFN-γ values from SARS-CoV-2-specific stimulations and the respective unstimulated controls. The geometric mean of the SI for both Ag1 and Ag2 at D35 had significantly declined by D215 (Figure 1C).

As we found positive SARS-CoV-2 N serology in 167 out of 600 individuals at D35, indicating a previous infection, we compared subpopulations that were N seronegative (D35 Nneg) and N seropositive (D35 Npos) at D35 to evaluate the effect of a prior SARS-CoV-2 infection on the level and the persistence of the immune response induced by the vaccination. SARS-CoV-2 S-specific IgG levels were significantly higher in the Npos group at both D35 and D215 compared to the Nneg group (Figure 2A). Importantly, whereas in the Nneg group we observed a more than 10-fold decline of the geometric mean of SARS-CoV-2 spike IgG levels at D215 relative to D35, the decrease in S-specific IgG response was significantly less in the Npos group (Figure 2D, Supplementary Table S1). Thus, SARS-CoV-2 S-specific IgG response was not only higher in the Npos group, but also persisted longer over time in individuals who experienced SARS-CoV-2 infection prior to vaccination. The geometric mean of anti-SARS-CoV-2 N antibodies in the Npos subjects at D35 decreased at D215 (Figure 2B), resulting in a reduction of about 65% relative to the values at D35 (Figure 2D, Supplementary Table S1). Of note, only 19 out of 167 D35 Npos individuals became N seronegative at D215 (Figure 2B).

Similar to SARS-CoV-2-specific S IgG response, we found significantly higher T cell responses for both Ag1 and Ag2 in the Npos group both at D35 and at D215 when compared to the Nneg group (Figure 2C). The SI values for both Ag1 and Ag2 decreased significantly from D35 to D215 regardless of whether they belonged to Nneg or Npos groups (Figure 2C). Interestingly, in contrast to anti-S IgG, we could not see any difference in the decline of T cell responses between Nneg and Npos groups when comparing the SI values at D215 relative to D35 (Figure 2D, Supplementary Table S1). T cell reactivity was more pronounced in the Npos group for both Ag1 and Ag2 at both D35 and D215 (Supplementary Figure S1C). At D35, 31.64% and 20.79 % of individuals did not respond to specific peptide stimulation in the Nneg group for Ag1 and Ag2, respectively. In contrast, only 17.96% and 13.77% of the Npos study subjects did not show reactivity upon stimulation with Ag1 and Ag2, respectively. At D215, still 65.87% (for Ag1) and 73.65% (for Ag2) of the participants of Npos groups remained reactive for antigenic stimulation. In the Nneg group reactivity decreased to 43.19% for Ag1 and 54.04% for Ag2, indicating that individuals with weaker reactivity might have lost this within the 6 months follow-up period (Supplementary Figure S1C). Of note, we could not find any sex-related differences in vaccine-induced SARS-CoV-2-specific immune responses in either the Nneg or Npos groups (Supplementary Figure S2).

As the age-related heterogeneity of the immune response has been documented after BNT162b2 vaccinations, we also investigated the correlation between age and immune parameters tested in the study population. With respect to anti-S IgG serum values, we found a weak negative correlation with the age in the D35 Nneg group at both D35 and D215. In contrast, in vaccinated study participants with prior SARS-CoV-2 infection (D35 Npos), a weak positive correlation between age and serum S IgG levels could be observed at both time points (Figure 3A). Similar to anti-S IgG, serum anti-N antibody levels correlated positively with age in the Npos group at both D35 and D215 (Figure 3B). However, we could not observe any correlation between age and T cell responses (SI) (Figure 3C).

We also correlated the different immune parameters with each other at both time points of sampling. In the Nneg group, the D35 values showed a positive correlation with the respective parameters measured at D215. Furthermore, we found a weak correlation between serum anti-S IgG levels with the SI for both Ag1 and Ag2 (Figure 4; Nneg). In the Npos group the D35 values showed a positive correlation for the respective parameters measured at D215. Between serum anti-S IgG and anti-N antibody levels a moderate correlation could be observed at both time points. Again, a weak positive correlation was found between both serum anti-S and anti-N antibody levels and T cell responses (Figure 4; Npos).

Lastly, we found 15 study participants out of the 600 with incident infection as determined by either becoming N seropositive by D215 or by a self-reported positive PCR between D35 and D215. A total of 13 out of the 15 belong to the D35 Nneg group, whereas 2 out of the 15 were N seropositive at the time point of study enrolment (D35 Npos) but reported a positive PCR during follow up, indicating re-infection in these study participants. This allowed us to compare immune responses with or without incident infection at least in the Nneg group (Figure 5, Supplementary Table S2). Of note, 13 out of the 15 participants with incident infection seroconverted for SARS-CoV-2 N antibodies (Figure 5B). Not surprisingly, the incident infection boosted SARS-CoV-2-specific immune responses as, in contrast to the significant reduction in the serum S IgG levels (Figure 5A) and the SI for both Ag1 and Ag2 (Figure 5C) observed in the Nneg group without incident infection between D35 and D215, we could not see any significant change in immune parameters in the Nneg group with incident infection. Of note, no difference in either antibody levels or T cell responses was seen at D35 between individuals with or without incident infections.

## 4. Discussion

Our study extends our knowledge about the duration of the immune response after BNT162b2 vaccination, particularly with regard to the T-cell response in a relatively large cohort of individuals from the general population. Importantly, both vaccinations were administered within one week, and thus investigations of SARS-CoV-2-specific immune responses were possible in a cohort exposed to the same virus variants and incidence rates during the follow up. The key findings of our study on the persistence of SARS-CoV-2-specific immune responses are as follows: (i) at 215 days after the second vaccination with BNT162b2, S-specific antibodies still persisted and, despite a significant reduction in the serum levels, could be detected in all individuals included in this analysis; (ii) cellular response to SARS-CoV-2 showed relatively large between-individual variations at day 35 and decreased over time but reactivity was still detectable in about half of the participants at day 215; and (iii) SARS-CoV-2 infections prior to vaccination or incident infections during the follow up boosted antibody levels and T cell responses at day 215 compared to study participants with no history of SARS-CoV-2 infection.

Consistent with previous studies, we found that two BNT162b2 vaccinations result in a robust antigen-specific IgG response in all investigated participants, which is in contrast to natural infections where antibody titer correlates with severity and seroconversion might even be absent, especially in some asymptomatic convalescent individuals [1,2,4,17]. Similarly to our results, robust SARS-CoV-2-specific T cell responses have already been documented in numerous studies after vaccinations and also after natural infections [18]. The waning of both SARS-CoV-2-specific humoral and cellular immunity over time is well documented and further supported with our results, which showed a significant reduction in both antibodies and T cell responses between D35 and D215 [5,6,9,19]. However, more than six months after the second immunization, we found detectable SARS-CoV-2-specific IgG levels in all of the participants. Importantly, SARS-CoV-2 infection before or after vaccination significantly boosted both S-specific IgG levels and T cell responses compared to two doses of the BNT162b2 vaccine alone. Since natural infections alone are thought to provide rather short-lived protection against SARS-CoV-2 reinfection [7,20], the importance of vaccination is highlighted regardless of the history of infection. In view of the emergence of new variants, this aspect is further emphasized as such hybrid immunity (natural infection followed by vaccination or vice versa) has been demonstrated to be the most efficient protection against the currently circulating omicron variants [21].

Whereas vaccine-induced neutralizing antibodies offer some protection against SARS-CoV-2 infections, SARS-CoV-2-specific T cells prevent severe outcome of COVID-19 disease. In contrast to the observed viral escape from neutralizing antibody response, T cell responses seem to be less affected by mutations characterized in different SARS-CoV-2 variants [22]. Thus, even if the mutations escape antibody neutralization, T cell activity, which is also present against highly mutated variants, might still protect from severe disease. In this context, it is important to note that T cell reactivity could still be detected 215 days after the second BNT162b2 vaccination in about 40-50 percent of participants with no history of previous infection. Moreover, the proportion of individuals with detectable T cell reactivity was clearly elevated in participants with natural infection prior to vaccination. Thus, a third immunization regardless of whether it is a natural infection or a further vaccine dose seems to be an advantage to prolong the persistence not only SARS-CoV-2-specific antibodies but also cellular responses and thereby possibly contributes to the reduction in the fatal outcome of COVID-19 even if antibody responses lose neutralizing capacity against new emerging variants [14].

Concerning the immunogenicity and persistence of SARS-CoV-2-specific immunity among the elderly, we found a weak negative correlation between age and the level of BNT162b2-induced spike-specific antibodies in individuals with no history of previous infection. The aging of immunity is documented to negatively influence vaccine-induced antibody response not only against SARS-CoV-2 but also other viruses such as influenza [23,24,25,26]. Similar to our results, Bates et al. recently documented that age negatively correlates with antibody response in vaccinated individuals without a history of infection [24]. Nevertheless, in the same study, no correlation of antibody response with age could be found in individuals with hybrid immunity [24]. However, our study included more individuals vaccinated twice with BNT162b2 with prior infection history, demonstrating a weak but significant positive correlation of both S- and N-specific antibody responses with age. Thus, on the one hand, the aging of immunity negatively impacts antibody responses after vaccination, and on the other hand, a greater disease severity associated with advanced age might result in an increase in humoral response [27]. The aging of immunity also impacts specific cellular responses, resulting in weaker responses against new infections and vaccinations [28]. An impaired functional SARS-CoV-2-specific T cell response after two doses of BNT162b2 vaccine has been documented in older people [23]. Similarly, a delayed antibody and T cell response to BNT162b2 vaccination has been found in the elderly, supporting a negative impact of age on vaccine-induced SARS-CoV-2-specific T cell responses [29]. We, however, could not see any correlation of age with T cell responses in our cohort. Both of the referred studies compare T cell immunity between younger individuals and elderly people with a median age over 80 years. This is in contrast to our study cohort, which includes vaccinated individuals up to an age of 80 years. Thus, our study implies a relative stable induction of T cell immunity in response to BNT162b2 vaccination until an age of 65–70 years. However, a significant drop of the T cell responsiveness over this age is obvious [23,29], which might be improved by a regular booster vaccination in this COVID-19-vulnerable older age group.

Our study has some limitations. We did not determine the neutralizing capacity of the antibodies induced by BNT162b2, which represents a crucial functional feature with respect to protection, especially against different variants. However, the neutralization capacity has been shown to correlate very well with antibodies induced by vaccination against SARS-CoV-2 S RBDs [30,31]. Importantly, neutralization capacity is highly dependent on the investigated variants [21,32]. Further, we only measured SARS-CoV-2-specific IgG but not IgA response; however, the latter is more important for the protection at mucosal surfaces [33]. Lastly, SARS-CoV-2-specific T cell responses were determined based on the IFN-γ production in response to specific peptide stimulation using QFN IGRA in whole blood, allowing us to screen the cellular response against the virus in a large number of study subjects. Nevertheless, a full characterization of T cell response with respect to the source of IFN-γ (CD4 or CD8 T cells), phenotypical and further functional analysis of T cells is missing.

## 5. Conclusions

Taken together, our study reveals sustained immunity after BNT162b2 prime/boost vaccination, including both the humoral and the cellular arms, which waned but was still detectable over 6 months in the majority of individuals. Importantly, SARS-CoV-2 infections prior to vaccination boosted both antibody and T cell responses, emphasizing the usefulness of vaccination even after a natural infection.

## Figures and Tables

**Table 1:** Demographic characteristics of the study cohort. D35 Nneg and D35 Npos are individuals who tested negatively and positively, respectively, for N-specific antibodies 35 day after the second BNT162b2 vaccination.

Age	Total (*n* = 600)		D35 Nneg (*n* = 433)		D35 Npos (*n* = 167)	
	Female (*n* = 379)	Male (*n* = 221)	Female (*n* = 279)	Male (*n* = 154)	Female (*n* = 100)	Male (*n* = 67)
min–max	16.79–79.28		16.79–79.28		17.17–75.64	
	16.79–79.28	17.7–78.67	16.79–79.28	17.7–78.67	17.17–74.05	18.98–75.64
mean (95% CI)	47.94 (46.83–49.05)		47.69 (46.40–48.99)		48.59 (46.42–50.77)	
	46.74 (45.33–48.15)	50.0 (48.23–51.78)	46.47 (44.85–48.10)	49.9 (47.78–52.03)	47.5 (44.62–50.38)	50.23 (46.89–53.57)
median (95% CI)	48.28 (46.03–50.17)		47.73 (45.02–49.71)		50.61 (45.99–54.12)	
	46.37 (42.83–49.48)	50.23 (47.73–52.10)	44.67 (42.41–49.0)	49.91 (47.26–52.47)	50.39 (42.19–54.27)	50.83 (44.24–56.20)

## Data Availability

Not applicable.

## Supplementary Materials

The following supporting information can be downloaded at: https://www.mdpi.com/article/10.3390/v14081642/s1. Figure S1: T cell responses followed two BNT162b2 vaccinations; Figure S2: No sex-related difference in serum levels for SARS-CoV-2-specific S and N antibodies and T cell responses based on SI calculated for both Ag1 and Ag2; Table S1: Relative response in D35 Nneg and Npos groups for S IgG, N Ig, Ag1, Ag2 and Mit calculated from values measured at D215 relative to D35; Table S2: Effect of an incident infection before D215 on the vaccine induced SARS-CoV-2-specific immune response in D35 Nneg and Npos groups.
