# Supplementary material for: Six-Month Follow-Up of Immune Responses after a Rapid Mass Vaccination against SARS-CoV-2 with BNT162b2 in the District of Schwaz/Austria
Source: Viruses. 2022 Jul 27;14(8):1642. doi: 10.3390/v14081642 (PMC9414611; doi:10.3390/v14081642)
Supplement: Supplementary file 1 [file viruses-14-01642-s001.zip › viruses-1827006-supplementary.pdf]

Six-Month Follow-Up of Immune Responses after a Rapid Mass Vaccination against SARS-CoV-2 with BNT162b2 in the District of Schwaz/Austria

Zoltán Bánki [1], Lisa Seekircher [2], Barbara Falkensammer [1], David Bante [1], Helena Schäfer [1], Teresa Harthaller [1], Janine Kimpel [1], Peter Willeit [2,3], Dorothee von Laer [1] and Wegene Borena [1]

[1] Institute of Virology, Medical University of Innsbruck, 6020 Innsbruck, Austria
[2] Clinical Epidemiology Team, Medical University of Innsbruck, 6020 Innsbruck, Austria
[3] Department of Public Health and Primary Care, University of Cambridge, CB2 1TN Cambridge, UK

Supplementary Figures

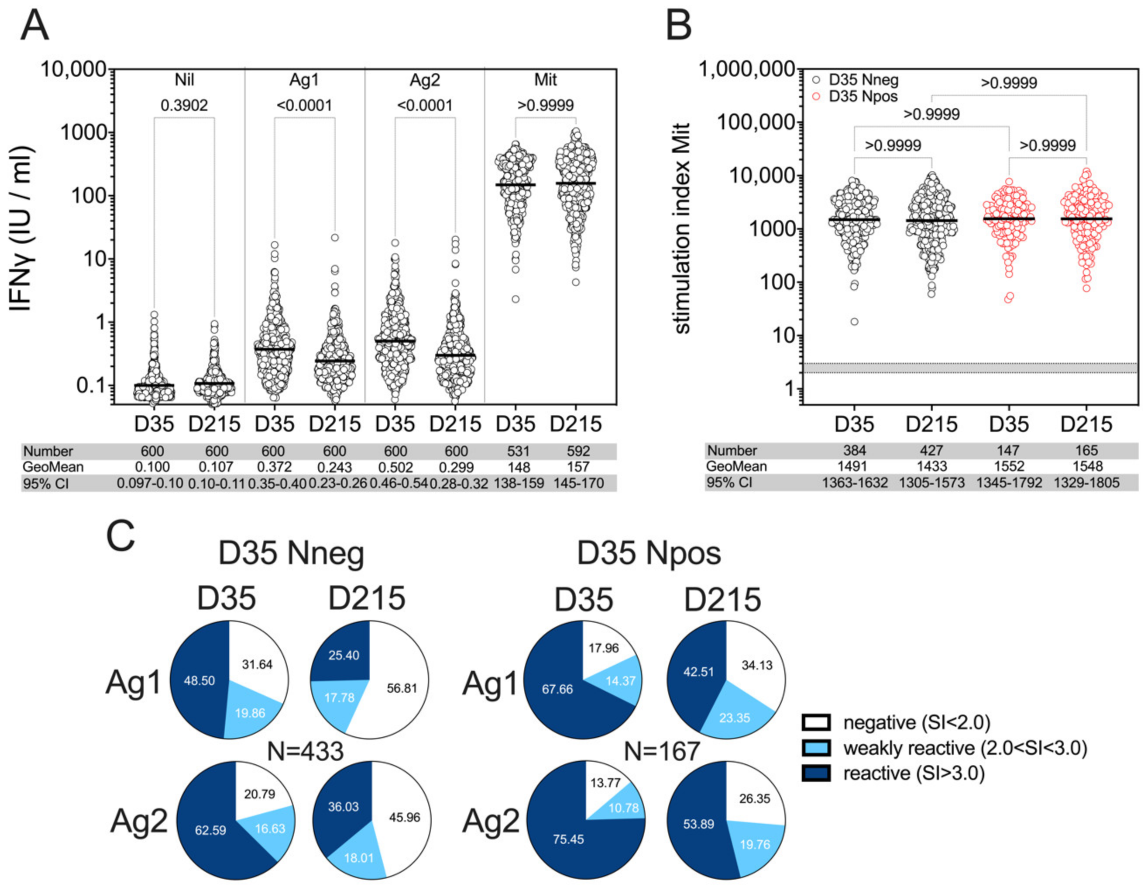

Supplementary Figure S1. T cell responses followed two BNT162b2 vaccinations. (**A**) IFNγ levels measured by ELISA in supernatants of heparinized whole blood stimulated for 24 hours in QFN tubes coated with two spike-derived peptide antigen pools (CD4 peptide pool Ag1 and CD4/CD8 peptide pool Ag2), and in QFN tubes for a negative control (Nil) and for a positive control (Mitogen). (**B**) No difference between D35 Nneg and D215 Npos groups in SI calculated after mitogen (Mit) stimulation. (**C**) T cell reactivity in the D35 Nneg and Npos groups considering SI values <2 as negative, 2 ≤ SI < 3 as weakly reactive and values ≥3 as reactive.

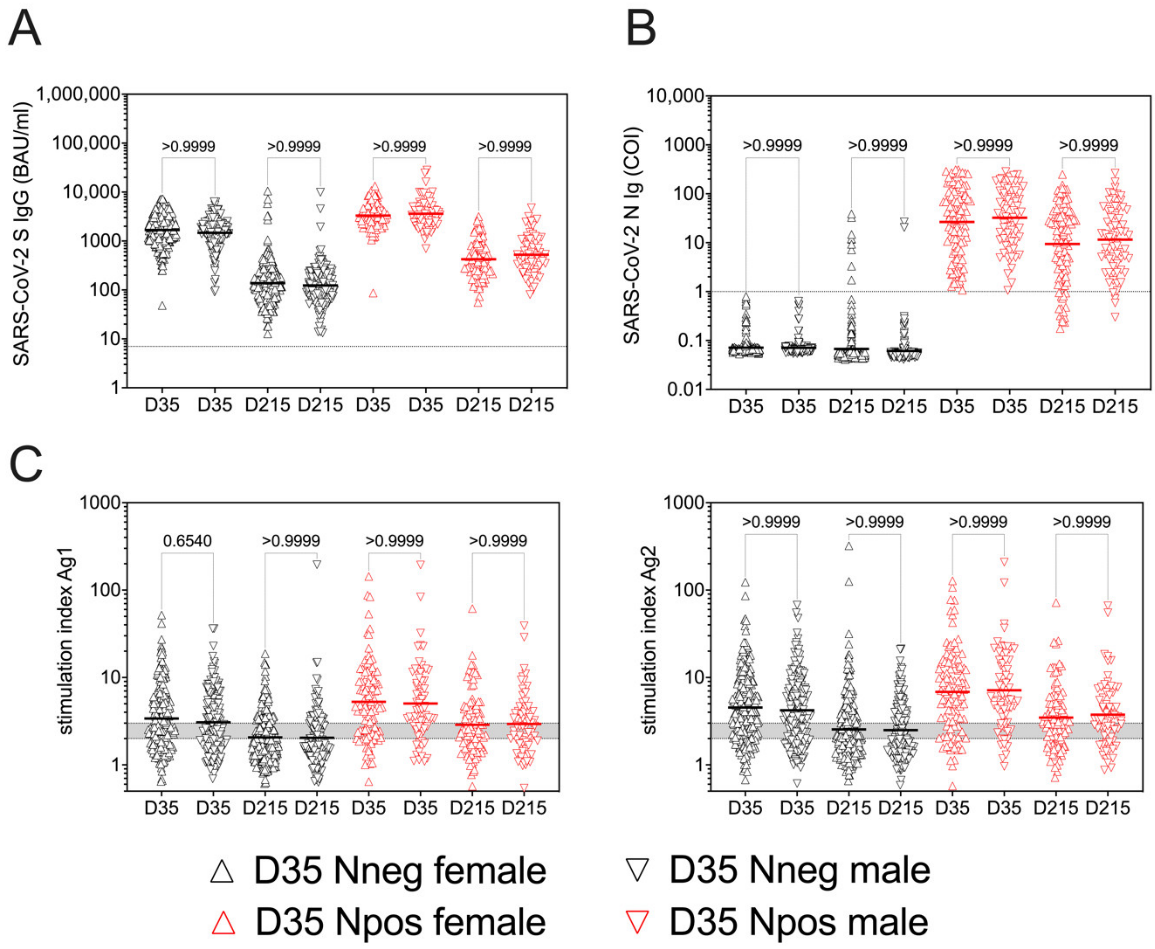

Supplementary Figure S2. No sex-related difference in serum levels for SARS-CoV-2-specific S (**A**) and N (**B**) antibodies and T cell responses based on SI calculated for both Ag1 and Ag2 (**C**).

Supplementary Tables

Supplementary Table S1. Relative response in D35 Nneg and Npos groups for S IgG, N Ig, Ag1, Ag2 and Mit calculated from values measured at D215 relative to D35.

	**S IgG**		**N IgG**		**Ag1**		**Ag2**		**Mit**	
	**D35 Nneg**	**D35 Npos**	**D35 Nneg**	**D35 Npos**	**D35 Nneg**	**D35 Npos**	**D35 Nneg**	**D35 Npos**	**D35 Nneg**	**D35 Npos**
Number	433	167	433	167	433	167	433	167	378	146
GeoMean	0.083	0.13	0.91	0.35	0.63	0.56	0.57	0.52	0.88	0.92
95% CI	0.08–0.09	0.13–0.14	0.85–0.99	0.32–0.39	0.59–0.67	0.51–0.62	0.53–0.62	0.46–0.58	0.81–0.95	0.80–1.1

Supplementary Table S2. Effect of an incident infection before D215 on the vaccine induced SARS-CoV-2-specific immune response in D35 Nneg and Npos groups. Serum levels of SARS-CoV-2 S- and N-specific antibodies as well as T cell response represented by stimulation index (SI) to Ag1 and Ag2 are shown in samples collected at D35 and D215 with or without incident SARS-CoV-2 infection.

		**D35 Nneg**		**D35 Nneg w/ Incident Infection**		**D35 Npos**		**D35 Npos w/ Incident Infection**	
		**D35 (N = 420)**	**D215 (N = 420)**	**D35 (N = 13)**	**D215 (N = 13)**	**D35 (N = 165)**	**D215 (N = 165)**	**D35 (N = 2)**	**D215 (N = 2)**
S IgG (BAU/ml)	GeoMean	1599	121.8	1592	1992	3445	464.0	2041	324.4
	95% CI	1491–1716	113.1–131.0	1060–2390	927.2–4277	3108–3818	404.7–532.1	n.a.	n.a.
N Ig (COI)	GeoMean	0.071	0.057	0.095	6.18	29.4	10.0	3.72	35.5
	95% CI	0.068–0.073	0.055–0.059	0.058–0.16	2.0–19.0	23.2–37.3	7.74–13.0	n.a.	n.a.
SI Ag1	GeoMean	3.27	2.03	3.37	3.27	5.20	2.91	3.4	2.0
	95% CI	3.03–3.53	1.90–2.16	2.21–5.13	2.41–4.44	4.44–6.08	2.58–3.29	n.a.	n.a.
SI Ag2	GeoMean	4.42	2.46	4.33	5.69	6.99	3.61	4.4	2.07
	95% CI	4.05–4.82	2.29–2.65	2.81–6.67	2.55–12.7	5.91–8.28	3.16–4.11	n.a.	n.a.
